# Sex differences in associations between multimorbidity and physical function domains among community-dwelling adults in Singapore

**DOI:** 10.1371/journal.pone.0197443

**Published:** 2018-05-14

**Authors:** Lixia Ge, Chun Wei Yap, Bee Hoon Heng

**Affiliations:** Health Services and Outcomes Research, National Healthcare Group Pte Ltd, Singapore, Singapore; Cardiff University, UNITED KINGDOM

## Abstract

**Objectives:**

The aims of the study were to identify the associations between multimorbidity and specific physical function domains among community-dwelling adults in Singapore, and to examine sex differences in the associations.

**Methods:**

This study was conducted using baseline data of 1,940 participants in the Population Health Index Survey conducted in the Central Region of Singapore from November 2015 to November 2016. Physical function was assessed using the Function Component of the Late-life Function and Disability Instrument and compared between men and women. Multiple linear regressions were conducted to examine associations between multimorbidity and different physical function domains for all participants, and in men and women separately.

**Results:**

The prevalence of multimorbidity in the study population was 35.0% for adults aged 21 years and above, with no differences between men and women. Multimorbidity was associated with reduced upper extremity function, basic and advanced lower extremity function, and overall function in men and women after adjusting for demographic factors. Multimorbidity had a stronger association with advanced lower extremity function and overall physical function in women than in men.

**Conclusions:**

The findings of this study indicate that multimorbidity is associated with physical function domains in men and women, and in particular advanced lower extremity for women. Effective community-based interventions need to be implemented to preserve physical function in individuals with multimorbidity to keep them functionally independent and physically active in the community. Additional focus on advanced lower extremity function for women is needed.

## Introduction

People living with multiple chronic diseases have become a common phenomenon because of therapeutic advances and increased longevity. Multimorbidity, the co-presence of two or more chronic medical conditions in an individual [1], requires complex care management and has become a large burden to the healthcare system and society [2–6]. Estimates of multimorbidity prevalence vary widely, ranging from 24.5% [7] to 98.5% [8]. It depends on countries, settings (primary care or community), sources of data (survey, administrative database, chart reviews or clinical evaluations), number and types of chronic diseases considered and individual characteristics like sex and age [8,9]. Although it is consistently reported in scientific literature that multimorbidity prevalence increases with age, more than half of the population with multimorbidity are younger than 65 years [10,11]. Hence, the increasing prevalence of multimorbidity in young and middle-aged adults should not be overlooked.

Limitations in physical function are great concerns to public health as they are highly associated with various negative health outcomes like decreased quality of life, increased risk of disability and level of dependency, increased health care utilization and costs [12]. Studies have shown that chronic diseases like stroke, heart diseases, chronic pulmonary diseases, osteoarthritis, depression and cognitive impairment can cause decline in physical function [13]. Furthermore, prior literature has documented the association between number of chronic diseases and poorer physical function in older adults [14,15]. While limited, a few large and rigorous studies in recent years (e.g. the Nurses’ Health Study II) have also reported lower physical function in young and middle-aged adults with multimorbidity [16,17]. Importantly, the cumulative impact of multimorbidity increases the likelihood of functional decline beyond the risk attributable to individual diseases [18,19]. However, the majority of these studies were conducted among older population and focused mainly on general physical function measured using physical health component of quality of life instruments, with few studies exploring the associations between multimorbidity and specific domains of physical function (i.e. upper and lower extremity function) among general community-dwelling adult population. Additionally, little is known about whether there is any sex difference with respect to the associations between multimorbidity and different domains of physical function, despite sex differences in physical performance and physical dysfunction were documented in literature [20,21]. Hence, the current study was conducted to examine the associations between multimorbidity and specific domains of physical function among community-dwelling men and women and to determine whether there are sex differences in the associations.

## Methods

### Data source and study population

This study was conducted as a cross-sectional analysis of baseline data derived from the Population Health Index (PHI) survey. This was a longitudinal household survey on the health of a representative sample of community-dwelling adult population (aged 21 and above) living in the Central Region of Singapore. Details of the study methodology have been previously described [22]. Briefly, eligible individuals (Singapore citizens or permanent residents, aged 21 years and above, having stayed in randomly selected household units for more than 6 months in the past year) were randomly selected using Kish grid tables [23] during house visits and underwent detailed structured interviews conducted by trained surveyors. Individuals were excluded from the survey if they were incapable of responding to the questionnaire due to mental health conditions, severe intellectual disability or cognitive impairment, or any communication issues (including language barriers, hearing loss, verbal aphasia following stroke) unless a proxy (usually a family member or caregiver who was most familiar with the individual’s condition) was available to give consent and provide the required information.

A total of 1,942 eligible individuals (including 17 proxy interviews) participated in the baseline PHI survey between November 2015 and November 2016. The response rate of the baseline PHI survey was 53.3%. Only participants with complete responses to the primary outcome measure of physical function (n = 1,940) were selected for the analyses.

The PHI survey was approved by the ethical committee of the National Healthcare Group Domain Specific Review Board (DSRB, Reference Number: 2015/00269). Written informed consent was obtained from individual participants in the survey.

### Measures

#### Physical function

The 32-item Function Component of the Late-life Function and Disability Instrument (LLFDI) [24] was used to measure physical function of the participants (S1 Appendix). The LLFDI was developed from the conceptual scheme of disablement to evaluate function and disability for community-dwelling older adults. The Function Component of the LLFDI evaluates self-reported difficulties in performing 32 routine physical activities of daily life which consist of three domains: upper extremity functioning (UEF, 7 items that reflect activities of the hands and arms), basic lower extremity functioning (BLEF, 14 items that reflect activities primarily involving standing, squatting, and fundamental walking activities), and advanced lower extremity functioning (ALEF, 11 items reflecting activities that involve a high level of physical ability and endurance) (S2 Appendix). Questions are phrased “How much difficulty do you have [*doing a particular activity*] without the help of someone else and without the use of any assistive walking device?”. Each question has five response options: “5 = None”, “4 = A little”, “3 = Some”, “2 = Quite a lot”, and “1 = Cannot do”. As a comprehensive measure that focuses on discrete actions or activities rather than narrow basic physical skills, the Function Component of the LLFDI is effective in capturing variations in function in the general population [24]. Multiple studies have evaluated the psychometric properties of the Function Component of the LLFDI and shown that it is a reliable and valid measure for physical function [25–27]. The LLFDI overall function and its three separate domains in the present study demonstrated high level of reliability (Cronbach's alpha of 0.973 for overall function and 0.897–0.961 for individual domains).

Scoring of the Function Component includes an overall function score and three separate earlier mentioned domain scores. The raw summary score of all 32 items and each domain was transformed based on a one-parameter Rasch model to a scaled score (0–100) in order to have the overall function score and all the domain scores on a similar metric [28]. Scores approaching 100 represent high levels in ability to perform discrete actions and activities without assistance while scores approaching 0 represent low levels in ability.

The proxies of the 17 participants who were incapable to report by themselves were asked to rate how they (the proxy) thought the participants would rate their own responses if able to communicate it.

#### Chronic diseases and multimorbidity

In this study chronic diseases referred to diseases that are irreversible and persistent through adulthood [17]. Seventeen chronic diseases (with similar diseases treated as one group) were used to determine multimorbidity. These were dyslipidemia, high blood pressure, diabetes, chronic kidney disease (CKD), heart attack / ischemic heart disease (IHD), heart failure, stroke / transient ischemic attack (TIA), asthma, chronic bronchitis / emphysema / chronic obstructive pulmonary disease (COPD), cancer, osteoarthritis / gout / rheumatoid arthritis (RA), osteoporosis, depression, anxiety disorder, schizophrenia, dementia / Alzheimer’s, and Parkinson’s disease. The diagnoses of these chronic diseases were obtained from two sources: 1) PHI survey data by asking the participants a specific question: “Have you ever been told to have any of these conditions *[the list of the 17 chronic diseases]* by a Western-trained doctor?”, and 2) the National Healthcare Group (NHG) Chronic Disease Management System (CDMS) database. This is a chronic disease registry within NHG that links administrative and key clinical data of patients with chronic diseases across the healthcare cluster in Singapore [29,30]. A total of 1,676 out of 1,940 participants (86.4%) in the PHI survey were identified in the CDMS database. A participant was considered to have a chronic disease if at least one of the two sources indicated the presence of that chronic disease, i.e. if a participant’s response to the survey question for a chronic disease was “No”, but the CDMS database showed that he/she had been diagnosed with that chronic disease, the participant was considered to have that chronic disease. Participants were stratified into three groups by number of chronic diseases: no chronic disease, 1 chronic disease, and 2 or more (2+) chronic diseases. Multimorbidity was defined as the presence of at least two of the 17 chronic diseases in this study.

#### Socio-demographic information

Socio-demographic information of the participants including age (categorized into 3 groups: 21–44, 45–64, and ≥65 years), sex, ethnicity (Chinese, Malay, Indian, and others), highest education level (no formal education, primary, secondary, and post-secondary and above), and smoking status was used in this study. Self-reported frequency of physical activities were captured using two questions: “How often do you take part in active recreation (e.g. bowling, golf, tennis, hiking, jogging, swimming, etc.)” and “How often do you take part in a regular fitness program (e.g. walking for exercise, stationary biking, weight lifting, or exercise classes, etc.)” with five response options for each question: “very often”, “often”, “once in a while”, “almost never”, and “never”.

### Statistical analysis

Sample weights were calculated according to a three-step procedure that included weights for the household, the household non-participant adjustment and the household member [22]. Descriptive analyses were conducted for socio-demographic characteristics with weighted mean and standard errors calculated for continuous variables, and unweighted frequencies and weighted percentages reported for categorical variables. Independent-samples t-tests or Chi-square (χ^2^) tests were conducted to assess differences in socio-demographic characteristics, presence of individual chronic diseases, number of chronic diseases and physical function domain scores between men and women. One-way analysis of variance (ANOVA) was used to determine the differences in physical function domains among adults with 0, 1 and 2+ chronic conditions.

Multiple linear regressions were conducted to examine the association between multimorbidity and overall function as well as individual domains of physical function. In each regression model, the dependent variable was overall function or individual physical function domain score, and the independent variable was multimorbidity, adjusted for demographics including age group, sex, ethnicity, highest education level and smoking status. Sex difference was examined by running the regressions separately for men and women, adjusted for the same covariates except sex. The regression coefficient (B) and standard error (SE) were reported. The significant level of difference between regression coefficients for the same physical function in men and women were tested using the z test [31]. Descriptive statistics were calculated using Statistical Package for Social Sciences (SPSS) version 18.0 (SPSS, Inc., Chicago, IL) and multiple linear regressions were performed using Stata/SE 12 for Windows. The result was considered significant if a *p* value was <0.05.

## Results

### Sample characteristics

The final study population comprised 1,940 participants. The weighted mean age of the study population was 51.4 years (standard deviation (SD):17.3 years, range: 21–97 years). More than half (56.1%) of the study population were women, and 78.3% were Chinese. The characteristics of the study population were summarized by sex in Table 1. The percentage of women who were current or past smokers (7.1%) was much lower than that of men (45.1%). Compared to women, participated in active recreation and regular fitness program was more frequent among men.

**Table 1:** Characteristics of the study population.

Variable	Total, n (%) (N = 1,940)	Men, n (%) (n = 859)	Women, n (%) (n = 1,081)	*p*-value (χ^2^ tests)
**Age group (Mean±SD)**	51.4±17.3	52.0±17.5	50.9±17.1	0.17
21–44	647 (36.7)	273 (35.0)	374 (38.0)	
45–64	776 (38.9)	354 (38.7)	422 (39.1)	
≥ 65	517 (24.4)	232 (26.3)	285 (22.9)	
**Ethnicity**				0.74
Chinese	1522 (78.3)	671 (77.8)	851 (78.7)	
Malay	154 (8.2)	68 (8.3)	86 (8.2)	
Indian	209 (10.9)	99 (11.6)	110 (10.4)	
Others	55 (2.5)	21 (2.2)	34 (2.8)	
**Highest education level**				**0.01**
No formal education	258 (12.6)	93 (9.9)	165 (14.8)	
Primary	251 (12.5)	115 (12.8)	136 (12.3)	
Secondary (sec)	600 (29.4)	261 (29.7)	339 (29.2)	
Post sec & above	831 (45.4)	390 (47.6)	441 (43.7)	
**Smoking status**				**<0.01**
Never smoked	1444 (76.2)	452 (54.9)	992 (92.8)	
Current smoker	258 (12.4)	212 (23.1)	46 (4.0)	
Former smoker	238 (11.4)	195 (22.0)	43 (3.1)	
**Participating in active recreation**				**<0.01**
Never	845 (42.1)	315 (34.5)	530 (48.0)	
Almost never	139 (7.2)	63 (7.2)	76 (7.3)	
Once in a while	388 (19.9)	159 (18.1)	229 (21.2)	
Often	396 (20.9)	210 (25.3)	186 (17.5)	
Very often	172 (10)	112 (14.9)	60 (6.1)	
**Participating in regular fitness program**				**<0.01**
Never	569 (28.7)	235 (26.0)	334 (30.8)	
Almost never	159 (7.5)	56 (5.9)	103 (8.8)	
Once in a while	360 (18.4)	145 (16.5)	215 (19.8)	
Often	518 (27.0)	244 (28.9)	274 (25.5)	
Very often	334 (18.4)	179 (22.7)	155 (15.0)	

*Note*: *Numbers in parentheses are weighted column %*.

### Prevalence of chronic diseases and multimorbidity

More than 55.0% of the study population had at least one chronic disease, and 35.0% had two or more chronic diseases. The average number of chronic diseases was 1.5 (SD: 1.9, range: 0–11) in men and 1.3 (SD: 1.7, range: 0–9) in women. There were 58.0% male and 53.4% female participants with at least one chronic disease. The prevalence of multimorbidity was 36.7% in men and 33.7% in women and these increased with increasing age in both sexes.

The most frequent chronic diseases were dyslipidemia, hypertension, and diabetes, with an overall prevalence of 35.7%, 30.4%, and 16.3%, respectively (Table 2). Dyslipidemia and hypertension were the top two common chronic diseases for both sexes. There were some differences in the pattern of chronic diseases between men and women. While men had significantly higher prevalence of hypertension, dyslipidemia, heart attack / IHD, heart failure, and chronic bronchitis / emphysema / COPD than women, their prevalence of osteoarthritis / gout / RA and depression was significantly lower (*p*<0.05).

**Table 2:** Sex- and age-specific prevalence of chronic diseases and multimorbidity, n (%).

Chronic condition	TOTAL (N = 1,940)	Men (n = 859)				Women (n = 1,081)			
		21–44	45–64	≥ 65	Total	21–44	45–64	≥ 65	Total
**Dyslipidemia**	711 (35.7)	28 (10.3)	144 (40.7)	166 (71.6)	338 (38.4)	20 (5.3)	160 (40.4)	193 (68.3)	373 (33.5)
**Hypertension**	625 (30.4)	26 (9.5)	125 (35.3)	160 (69.0)	311 (34.9)	11 (1.7)	108 (26.3)	195 (69.5)	314 (26.9)
**Diabetes**	317 (16.3)	13 (4.8)	58 (16.4)	80 (34.5)	151 (18.1)	19 (4.3)	66 (16.9)	81 (29.3)	166 (15.0)
**Osteoarthritis /gout /RA**	329 (15.5)	11 (4.0)	53 (15.0)	74 (31.9)	138 (14.6)	15 (3.1)	72 (16.7)	104 (36.9)	191 (16.3)
**Asthma**	133 (7.1)	24 (8.8)	14 (4.0)	14 (6.0)	52 (6.0)	41 (11.4)	30 (7.3)	10 (4.0)	81 (8.0)
**CKD**	109 (5.5)	0 (0)	11 (3.1)	46 (19.8)	57 (6.6)	2 (0.2)	12 (2.8)	38 (14.5)	52 (4.6)
**Osteoporosis**	115 (5.1)	0 (0)	8 (2.3)	20 (8.6)	28 (2.5)	3 (0.7)	28 (6.3)	56 (19.3)	87 (7.2)
**Heart attack/ IHD**	94 (4.7)	0 (0)	23 (6.5)	39 (16.8)	62 (7.2)	0 (0)	5 (1.4)	27 (9.6)	32 (2.8)
**Stroke/TIA**	90 (4.2)	0 (0)	10 (2.8)	33 (14.2)	43 (4.8)	0 (0)	17 (4.5)	30 (8.5)	47 (3.7)
**Cancer**	85 (4.1)	0 (0)	10 (2.8)	27 (11.6)	37 (4.0)	1 (0)	22 (5.6)	25 (8.5)	48 (4.2)
**Depression**	69 (3.3)	6 (2.2)	9 (2.5)	5 (2.2)	20 (2.1)	12 (2.7)	23 (4.9)	14 (5.2)	49 (4.2)
**Heart failure**	51 (2.6)	0 (0)	10 (2.8)	21 (9.1)	31 (3.8)	0 (0)	6 (1.4)	14 (5.2)	20 (1.7)
**Anxiety disorder**	50 (2.4)	7 (2.6)	8 (2.3)	6 (2.6)	21 (2.5)	2 (0.2)	16 (3.1)	11 (4.4)	29 (2.3)
**Chronic bronchitis /emphysema/COPD**	32 (1.5)	1 (0.4)	6 (1.7)	15 (6.5)	22 (2.5)	2 (0.2)	2 (0.7)	6 (2.0)	10 (0.8)
**Schizophrenia**	19 (1.0)	3 (1.1)	3 (0.8)	1 (0.4)	7 (0.7)	1 (0.5)	4 (0.9)	7 (2.8)	12 (1.2)
**Dementia/ Alzheimer’s**	19 (1.0)	0 (0)	1 (0.3)	6 (2.6)	7 (0.9)	0 (0)	2 (0.5)	10 (3.6)	12 (1.0)
**Parkinson’s disease**	9 (0.4)	0 (0)	1 (0.3)	5 (2.2)	6 (0.6)	0 (0)	1 (0.5)	2 (0.8)	3 (0.3)
**Number of chronic diseases**									
0	835 (44.6)	191 (70.0)	135 (38.1)	26 (11.2)	352 (42.1)	285 (77.3)	160 (36.8)	38 (12.4)	483 (46.6)
1	390 (20.4)	60 (22.0)	93 (26.3)	28 (12.1)	181 (21.3)	62 (16.6)	108 (26.2)	39 (13.3)	209 (19.7)
Multimorbidity (2+)	715 (35.0)	22 (8.1)	126 (35.6)	178 (76.7)	326 (36.7)		27 (6.0)	154 (36.9)	208 (74.3)	389 (33.7)

The top three combinations of chronic diseases in the study population were hypertension and dyslipidemia (4.8%), diabetes, hypertension and dyslipidemia (2.7%), hypertension, dyslipidemia and osteoarthritis / gout / RA (1.5%). Men had higher rate of co-presence of hypertension and dyslipidemia (5.6%) than women (4.2%).

### Physical function domains

As shown in Table 3, both men and women reported lower scores for BLEF and ALEF than UEF. Compared to men, women reported significantly lower scores in overall function and all three domains of physical function (*p*<0.01). With increasing age, both men and women reported decline in overall function and all three physical function domains, and the decline in ALEF was most predominant.

**Table 3:** Physical function domain scores (mean ± SD) by sex and age group.

Physical function domain	TOTAL (N = 1,940)	Men (n = 859)				Women (n = 1,081)			
		21–44	45–64	≥ 65	Total	21–44	45–64	≥ 65	Total
UEF	84.7±17.8	99.8±2.1	98.5±7.1	90.8±18.4	96.9±11.1	98.4±8.8	96.8±9.3	87.7±17.4	95.3±12.2
BLEF	96.0±11.8	99.6±2.7	96.7±10.0	82.8±23.5	94.1±15.2	98.3±9.1	93.5±13.1	77.7±21.2	91.7±16.2
ALEF	92.7±15.9	96.1±9.2	87.8±16.7	62.6±27.8	84.1±22.8	93.0±14.3	78.6±19.2	53.7±24.5	78.4±24.0
Overall	80.9±23.7	96.7±7.4	89.9±13.3	71.3±20.4	87.4±17.3		93.7±12.3	82.4±14.8	64.6±15.8	82.6±17.9

*Note*: *UEF = upper extremity function; BLEF = basic lower extremity function; ALEF = advanced lower* extremity function. *P<0*.*05 for all Independent-sample t-tests for UEF*, *BLEF*, *ALEF and overall function between men and women in every age group*, *except for UEF between men and women aged ≥65 years*.

### Associations between multimorbidity and individual physical function domains

**Fig 1:**
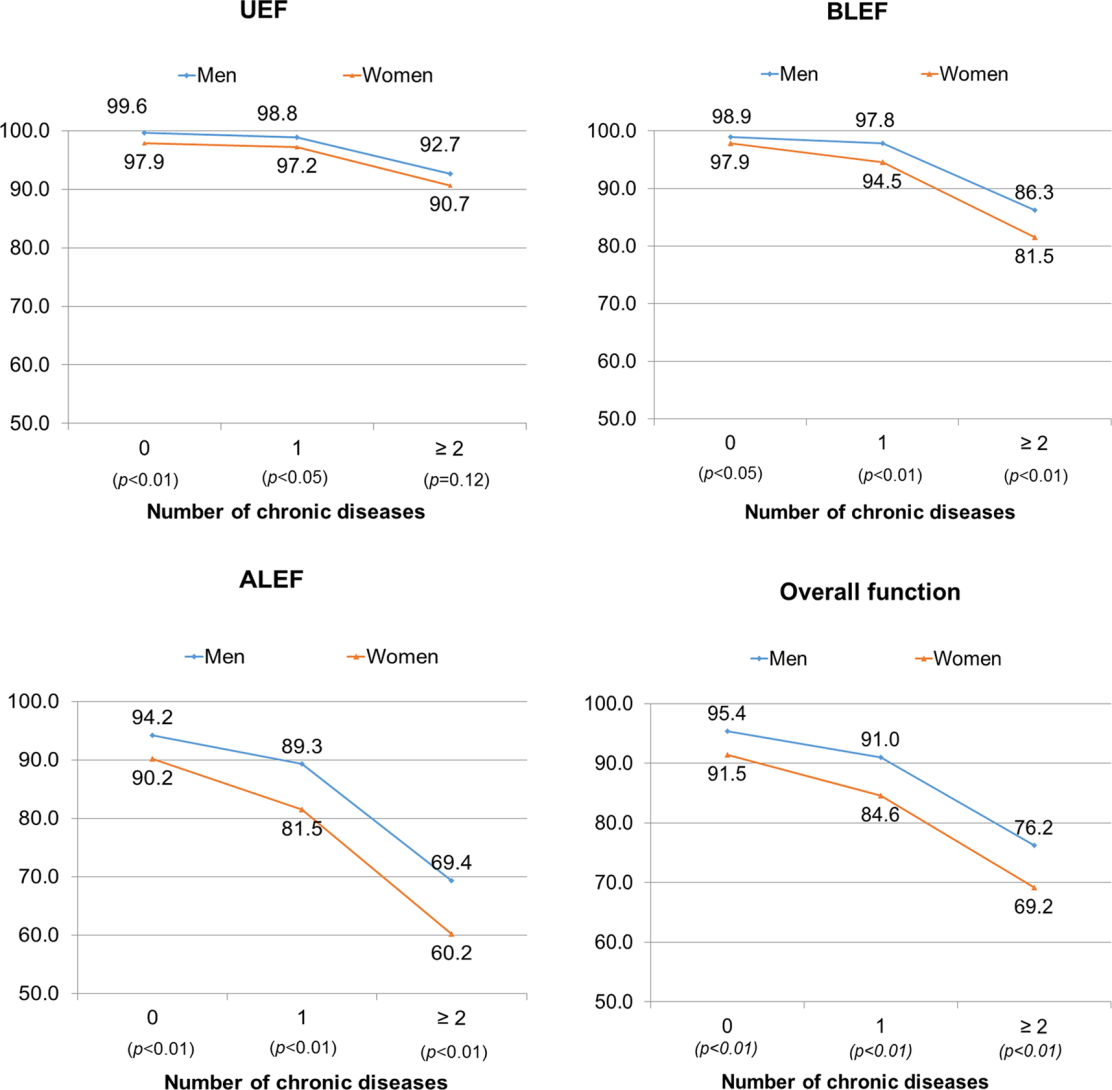
Sex differences in physical function domains by number of chronic diseases.

Fig 1 showed that with the increase in number of chronic diseases, there were significant decreases in overall function and individual domains of physical function for both men and women, of which ALEF had the most substantial decrease, followed by BLEF. Women consistently reported poorer BLEF and ALEF (*p*<0.05, Fig 1). However, while there was significant sex difference in UEF for those with zero or one chronic disease, the difference was not significant for those with two or more chronic diseases (*p* = 0.12).

The linear regressions for individual physical function domains for all participants showed that having one chronic condition was negatively associated with ALEF (B = -3.54, SE = 1.11) and overall function (B = -2.87, SE = 0.82). Multimorbidity was consistently associated with lower scores of UEF (B = -4.27, SE = 0.66), BLEF (B = -8.79, SE = 0.82), ALEF (B = -14.27, SE = 1.09) and overall function (B = -10.95, SE = 0.81). Additionally, being a woman was associated with lower scores in all physical function domains (Table 4).

**Table 4:** Associations between multimorbidity and physical function domains using multiple linear regressions.

Variables	UEF		BLEF		ALEF		Overall function	
	B	SE	B	SE	B	SE	B	SE
**Number of chronic diseases (Ref: No disease)**								
1	-0.05	0.67	-0.98	0.83	-3.54*	1.11	-2.87*	0.82
2+	-4.27*	0.66	-8.79*	0.82	-14.27*	1.09	-10.95*	0.81
**Age group (Ref:21–44)**								
45–64	0.41	0.67	0.005	0.83	-3.70*	1.10	-3.12*	0.82
≥ 65	-4.73*	0.90	-8.74*	1.11	-18.91*	1.49	-13.95*	1.10
**Women (Ref: Men)**	-1.67*	0.56	-2.73*	0.69	-6.62*	0.92	-5.35*	0.68
**Ethnicity (Ref: Chinese)**								
Malay	-1.35	0.92	-4.12*	1.14	-6.76*	1.52	-5.15*	1.12
Indian	-0.44	0.80	-2.52*	0.98	-4.02*	1.31	-2.68*	0.97
Others	-3.27*	1.48	-3.64*	1.83	-6.08*	2.44	-4.69	1.80
**Highest education (Ref: No formal education)**								
Primary	4.35*	0.97	5.32*	1.20	7.59*	1.60	4.97*	1.18
Secondary	5.41*	0.85	8.93*	1.05	12.40*	1.41	8.68*	1.04
Post-secondary	5.65*	0.95	9.25*	1.17	15.93*	1.56	11.45*	1.15
**Smoking status (Ref: Never smoked)**								
Never smoked	1.41	0.80	1.25	0.99	0.44	1.32	0.62	0.98
Former smoker	-1.49	0.81	-2.35*	1.00	-2.59	1.33	-1.41	0.98

*Note*: *UEF = upper extremity function; BLEF = basic lower extremity function; ALEF = advanced lower extremity function*, *B = regression coefficient*, *SE = standard error*

*p<0.*05*.

Table 5 below shows the multiple linear regression results of the associations between multimorbidity and individual physical function domains stratified by sex. Having one chronic disease was inversely associated with ALEF (B = -4.68, SE = 1.49) and overall function (B = -3.61, SE = 1.09) in women only. Multimorbidity was significantly associated with the decline in upper and lower extremity function in both men and women. The comparison between respective regression coefficients showed that multimorbidity had stronger association with ALEF (z-score = 1.91, *p*<0.01) and overall function (z-score = 1.60, *p* = 0.02) in women than in men.

**Table 5:** Sex differences in associations between multimorbidity and physical function domains using multiple linear regressions.

Variables	Men								Women							
	UEF		BLEF		ALEF		Overall function		UEF		BLEF		ALEF		Overall function	
	B	SE	B	SE	B	SE	B	SE	B	SE	B	SE	B	SE	B	SE
**Number of chronic diseases (Ref: no condition)**																
1	0.16	0.95	0.25	1.23	-1.91	1.67	-1.82	1.25	-0.36	0.95	-2.05	1.13	-4.68*	1.49	-3.61*	1.09
2+	-4.08*	0.94	-6.69*	1.23	-11.72*	1.66	-9.36*	1.25	-4.42*	0.92	-10.40*	1.10	-16.07*	1.45	-12.07*	1.06
**Age group (Ref:21–44)**																
45–64	0.07	0.93	-0.24	1.20	-2.62	1.63	-2.44*	1.22	0.88	0.98	0.17	1.16	-4.57*	1.54	-3.62*	1.12
≥ 65	-4.94*	1.25	-9.67*	1.63	-20.00*	2.21	-14.96*	1.66	-4.16*	1.30	-7.85*	1.54	-18.03*	2.04	-13.09*	1.50
**Ethnicity (Ref: Chinese)**																
Malay	-4.92*	1.33	-6.41*	1.73	-6.47*	2.34	-5.11*	1.76	1.26	1.27	-2.37	1.52	-6.87*	2.01	-5.02*	1.47
Indian	-0.98	1.11	-2.74	1.44	-4.04*	1.94	-2.95*	1.46		0.02	1.13	-2.43	1.35	-4.15*	1.78	-2.51	1.30
Others	-3.40	2.27	-2.91	2.95	-7.28	4.00	-5.20	3.00	-2.87	1.96	-3.92	2.33	-5.49	3.08	-4.51*	2.25
**Highest education (Ref: No formal education)**																
Primary	2.27	1.44	1.88	1.87	3.40	2.53	2.27	1.90	5.68*	1.31	7.45*	1.56	10.39*	2.07	6.76*	1.51
Secondary	3.00*	1.27	5.23*	1.65	8.95*	2.23	6.55*	1.67		11.13*	1.39	11.08*	1.39	14.56*	1.84	10.06*	1.35
Post-secondary	2.59	1.38	4.96*	1.79	11.40*	2.42	8.46*	1.82		11.85*	1.57	11.99*	1.56	18.64*	2.07	13.29*	1.52
**Smoking status (Ref: Never smoked)**																
Current smoker	1.64	0.91	1.63	1.18	0.13	1.60	0.19	1.20		-1.79	2.03	11.08*	1.39	-2.40	2.69	-0.28	1.97
Former smoker	-2.26*	0.91	-3.06*	1.18	-3.62*	1.60	-1.93	1.20		-1.47	2.07	11.99*	1.56	-1.34	2.74	-1.28	2.01

*Note*: *UEF = upper extremity function; BLEF = basic lower extremity function; ALEF = advanced lower extremity function*, *B = regression coefficient*, *SE = standard error*

*p<0.*05*.

## Discussion

This study showed that multimorbidity was associated with reduced upper extremity function, basic and advanced lower extremity function, and overall function measured using self-reported LLFDI in community-dwelling adults. The associations of multimorbidity with advanced lower extremity function and overall physical function were stronger in women than in men.

The estimated prevalence of multimorbidity in this study was 35.0% for adults aged 21 years and above and 68.2% for those aged 60 years and above. This is higher than 16.3% [32] and 51.5% [6] for the respective population reported in other local studies, and is similar to 30% reported among Spanish general population aged 20 years and above [33]. Because men had higher prevalence of the top three common chronic diseases (including hypertension, dyslipidemia and diabetes), the proportion of men having multimorbidity in the present study was slightly higher than that of women, though this is not statistically significant. This is contrary to the findings of some European studies which found that multimorbidity was more prevalent in older women than in older men [34,35]. This may be due to the differences in types and/or prevalence of the chronic diseases included and the age structure for men and women.

The negative association between multimorbidity and individual domains of physical function among community-dwelling adults in this study is in concordance with that found in one previous study in older individuals [36]. This can be explained by physical activity reduction, muscle mass and strength decrease, or physiological impairment caused by chronic diseases [36–39]. As reported by Melissa et al.[17], younger adults with multimorbidity also reported lower physical function, the significant associations after controlling for age group and other covariates in the study also indicates that multimorbidity’s negative impact on physical function is independent of aging and other demographics. Compared to UEF (e.g., unscrewing the lid of an unopened jar, pouring water from a large pitcher, holding a full glass of water, etc.), LEF especially ALEF (e.g. going up and down three flights of stairs, hiking a couple of kilometers on uneven surfaces, running 800 meters or more, etc.) declines more substantially with increase in number of chronic diseases in both men and women. A possible reason is that skeletal muscle dysfunction, a combination of reduced muscle strength and endurance, is one of the major secondary impairments associated with pathological changes caused by chronic diseases which predominantly affects the lower extremity muscles, while the muscle function is better preserved in the upper extremity [40–42]. Other possible reasons could include the type / prevalence of chronic diseases included (e.g. Parkinson’s disease affects UEF but is less prevalent) and potential measurement issues (UEF is assessed using fewer items than LEF and those upper extremity activities require relatively less energy and endurance).

Consistent with previous studies in middle-aged and older adults in Denmark and Russia [43], and Germany [44] using SF-36 physical functioning subscale, the results in this study also showed that women reported poorer physical function than men. The sex difference was most obvious in ALEF with a mean score difference of 4.5. This is probably due to the differences in body composition between men and women [20]. Men are generally superior in muscle strength, muscle endurance, whole-body endurance, and walking ability compared to women [45]. Despite the difference in chronic diseases prevalence, consistent associations between multimorbidity and individual domains of physical function were observed in both men and women. Compared to men, multimorbidity had stronger association with ALEF in women, which suggests that women’s ALEF is more vulnerable to multimorbidity than men’s.

Furthermore, as whole-body and muscle endurance, muscle strength and walking ability decrease with age [45–46], the decline of various physical functions with age in both men and women were also observed in the present study. Similar to the findings reported in an European study [47], lower education level was also associated with poorer physical function for both sexes. Furthermore, Malay males reported poorer physical function than Chinese males, which is consistent with the current ethnic differences in physical health in Singapore [48].

As a population-based health survey, the PHI survey questionnaire was administered to a representative study population, the findings of the study are generalizable to the general adult population in the Central Region of Singapore. However, there are a few limitations in this study. Firstly, self-reporting of chronic diseases may lead to an underestimation of the prevalence of chronic diseases. This risk was partially mitigated through the use of a chronic disease registry. However, as the CDMS database contains only patients who were seen in NHG institutions, participants whose chronic diseases were treated at other institutions or private general practitioners are still subjected to measurement error introduced by self-report. Secondly, using a simple disease count may not be sufficient to represent the full association between multimorbidity and physical function [17]. This is because a simple disease count does not take into account the severity of individual chronic diseases contributing to physical function decline and fails to identify any specific interactions of different chronic diseases that are driving physical function impairment. The use of complex approaches to measure multimorbidity (e.g. severity-weighted multimorbidity index and cluster analysis) may provide deeper insights on the association between multimorbidity and physical function. Finally, the cross-sectional nature of the present study prevents identification of causal relationships between chronic diseases and decline in physical function.

## Conclusions

The findings of this study show that multimorbidity is associated with individual domains of physical function in men and women, particularly among older women. Considering the high prevalence of multimorbidity among community-dwelling adults and its substantial association with different domains of physical function, healthcare system needs to take effective community-based interventions to preserve physical function in individuals with multimorbidity in order to keep them functionally independent and physically active in the community. Additional focus on advanced lower extremity function for women is needed.

## Supporting information

S1 Appendix 32-item Function Component of the Late-life Function and Disability Instrument (LLFDI). (PDF)

S2 Appendix Items used to assess specific physical function domains in the Function Component of the LLFDI. (PDF)
