# Supplementary material for: Sex differences in associations between multimorbidity and physical function domains among community-dwelling adults in Singapore
Source: PLoS One. 2018 May 14;13(5):e0197443. doi: 10.1371/journal.pone.0197443 (PMC5951575; doi:10.1371/journal.pone.0197443)
Supplement: S1 Appendix — (PDF) [file pone.0197443.s001.pdf]

S1 Appendix. 32-item Function Component of the Late-life Function and Disability Instrument (LLFDI)

	**How much difficulty do you have…?** **(Remember, this is without the help of someone else and without the use of any assistive walking device.)**	**5) None**	**4) A Little**	**3) Some**	**2) Quite a Lot**	**1) Cannot do**
1.	Unscrewing the lid off a previously unopened jar without using any devices	☐	☐	☐	☐	☐
2.	Going up and down a flight of stairs using a handrail	☐	☐	☐	☐	☐
3.	Putting on and taking off long pants (including managing fasteners)	☐	☐	☐	☐	☐
4.	Running 800 meters or more	☐	☐	☐	☐	☐
5.	Using common utensils for preparing meals (e.g., can opener, potato peeler, or sharp knife)	☐	☐	☐	☐	☐
6.	Holding a full glass of water in one hand	☐	☐	☐	☐	☐
7.	Walking 1.6 kilometres, taking rests as necessary	☐	☐	☐	☐	☐
8.	Going up & down a flight of stairs without using a handrail	☐	☐	☐	☐	☐
9.	Running a short distance, such as to catch a bus	☐	☐	☐	☐	☐
10.	Reaching overhead while standing, as if to pull a light cord	☐	☐	☐	☐	☐
11.	Sitting down in and standing up from a low, soft couch	☐	☐	☐	☐	☐
12.	Putting on and taking off a coat or jacket	☐	☐	☐	☐	☐
13.	Reaching behind your back as if to put a belt through a belt loop	☐	☐	☐	☐	☐
14.	Stepping up and down from a curb	☐	☐	☐	☐	☐
15.	Opening a heavy, outside door	☐	☐	☐	☐	☐
16.	Rip open a package of snack food (e.g. cellophane wrapping on crackers) using only your hands	☐	☐	☐	☐	☐
17.	Pouring from a large pitcher	☐	☐	☐	☐	☐
18.	Getting into and out of a car/taxi (sedan)	☐	☐	☐	☐	☐
19.	Hiking a couple of kilometres on uneven surfaces, including hills	☐	☐	☐	☐	☐

	How much difficulty do you have…? **<u>(Remember, this is without the help of someone else and without the use of any assistive walking device.)</u>**	5) None	4) A Little	3) Some	2) Quite a Lot	1) Cannot do
20.	Going up and down 3 flights of stairs using a handrail	☐	☐	☐	☐	☐
21.	Picking up a kitchen chair and moving it, in order to clean	☐	☐	☐	☐	☐
22.	Using a step stool to reach into a high cabinet	☐	☐	☐	☐	☐
23.	Making a bed, including spreading and tucking in bed sheets	☐	☐	☐	☐	☐
24.	Carrying something in both arms while climbing a flight of stairs (e.g. laundry basket)	☐	☐	☐	☐	☐
25.	Bending over from a standing position to pick up a piece of clothing from the floor	☐	☐	☐	☐	☐
26.	Walking around one floor of your home, taking into consideration thresholds, doors, furniture, and a variety of floor coverings	☐	☐	☐	☐	☐
27.	Getting up from the floor (as if you were laying on the ground)	☐	☐	☐	☐	☐
28.	Washing dishes, pots, and utensils by hand while standing at sink	☐	☐	☐	☐	☐
29.	Walking several blocks	☐	☐	☐	☐	☐
30.	Taking a 1.6 kilometres, brisk walk without stopping to rest	☐	☐	☐	☐	☐
31.	Stepping on and off a bus	☐	☐	☐	☐	☐
32.	Walking on a slippery surface outdoors	☐	☐	☐	☐	☐
