# Supplementary material for: Sex differences in associations between multimorbidity and physical function domains among community-dwelling adults in Singapore
Source: PLoS One. 2018 May 14;13(5):e0197443. doi: 10.1371/journal.pone.0197443 (PMC5951575; doi:10.1371/journal.pone.0197443)
Supplement: S2 Appendix — (PDF) [file pone.0197443.s002.pdf]

S2 Appendix. Items used to assess specific physical function domains in the Function Cmponent of the LLFDI.

Upper extremity function (7 items)	Basic lower extremity function (14 items)	Advanced lower extremity function (11 items)
• Unscrewing the lid off a previously unopened jar<br>• Putting on and taking off long pants<br>• Using common utensils for preparing meals<br>• Holding a full glass of water<br>• Reaching behind your back<br>• Ripping open package of snack food<br>• Pouring from a large pitcher	• Going up and down a flight of stairs, using handrail<br>• Reaching overhead while standing<br>• Sitting down in and standing up from low soft couch<br>• Putting on and taking off a coat<br>• Stepping up and down from a curb<br>• Opening a heavy outside door<br>• Getting into and out of a car<br>• Picking up a kitchen chair<br>• Using a step stool<br>• Making a bed<br>• Bending over from a standing position<br>• Walking around one floor of home<br>• Washing dishes, pots, and utensils while standing<br>• Stepping on and off a bus	• Running 1/2 mile or more<br>• Walking 1 mile, taking rests as necessary<br>• Going up & down a flight of stairs, without a handrail<br>• Running a short distance, such as to catch a bus<br>• Hiking a couple of miles<br>• Going up & down 3 flights, with handrail<br>• Carrying something in both arms while climbing stairs<br>• Getting up from the floor<br>• Walking several blocks<br>• Taking a 1 mile brisk walk without stopping<br>• Walking on a slippery surface outdoors
